# Comparison of the Conventional and Electroenhanced Direct-Immersion Solid-Phase Microextraction for Sampling of Nicotine in Biological Fluids of the Human Body

**DOI:** 10.3390/molecules23051171

**Published:** 2018-05-14

**Authors:** Sana Abdolhosseini, Ali Reza Ghiasvand, Nahid Heidari

**Affiliations:** Department of Chemistry, Lorestan University, P.O. Box 465, Khorramabad 44316-68151, Iran; sana_abdolhoseiny@yahoo.com (S.A.); nheidari44@yahoo.com (N.H.)

**Keywords:** direct-immersion SPME, electroenhanced SPME, nicotine, plasma, urine

## Abstract

A stainless steel fiber was made porous and adhesive by platinization and then coated by nanostructured polypyrrole (PPy), using an appropriate electrophoretic deposition (EPD) method. The morphological surface structure and functional groups of the PPy-coated fiber were studied using SEM (Scanning electron microscope) instrument. The prepared fiber was used for comparison of direct immersion (DI) and electroenhanced direct immersion solid-phase microextraction (EE-DI-SPME) of nicotine in human plasma and urine samples followed by gas chromatography flame ionization detector (GC-FID) determination. The effects of the influential experimental parameters on the efficiency of the DI-SPME and EE-DI-SPME methods, including the pH and ionic strength of the sample solution, applied Direct current (DC) voltage, extraction temperature and time and stirring rate, were optimized. Under the optimal conditions, the calibration curves for the DI-SPME-GC-FID and EE-DI-SPME-GC-FID methods were linear over the ranges of 0.1–10.0 μg mL^−1^ and 0.001–10.0 μg mL^−1^, respectively. The relative standard deviations (RSDs, *n* = 6) were found to be 6.1% and 4.6% for the DI and EE strategies, respectively. The LODs (limit of detection) of the DI-SPME-GC-FID and EE-DI-SPME-GC-FID methods were found to be 10 and 0.3 ng mL^−1^, respectively. The relative recovery values (for the analysis of 1 µg mL^−1^ nicotine) were found to be 91–110% for EE-DI-SPME and 75–105% for DI-SPME. The enrichment factors for DI-SPME and EE-DI-SPME sampling were obtained as 38,734 and 50,597, respectively. The results indicated that EE-SPME was more efficient for quantitation of nicotine in biological fluids. The developed procedure was successfully carried out for the extraction and measurement of nicotine in real plasma and urine samples.

## 1. Introduction

During the last two decades, chemical analysis strategies were directed toward green chemistry norms, and consequently, solvent-free sampling methods have been placed at the center of attention [1]. As a result, the development of green and sustainable analysis procedures is currently an interesting multidisciplinary topic in a broad spectrum of sciences, including chemical analysis, environmental monitoring, biochemistry and medicinal chemistry [2]. Following these principals, one of the most important steps in analytical scale separation has been taken by introducing solid-phase microextraction (SPME), as a fast, solvent-free, easy-to-operate and efficient sampling strategy. In SPME, the separation and preconcentration processes have been merged into one step [3]. It reduces the expenses, waste, steps and time of analyses and can be easily automated and used for in vitro/in vivo biological monitoring studies [4].

SPME is coupleable with the most commonly-used chromatographic techniques such as gas chromatography (GC), high-performance liquid chromatography (HPLC) and supercritical fluid chromatography (SFC) [5]. It can be applied in three sampling modes including direct-immersion (DI-SPME), headspace (HS-SPME) and membrane-protected SPME (MP-SPME) [6]. Different variables that affect analyte uptake, in SPME studies, such as sampling mode, the coating’s chemistry, sample matrix complexity and the physicochemical properties of analytes, should be considered [7].

Headspace is the most used mode of SPME, which extracts analytes from the headspace of the sample, without contact with the sample matrix [8]. Nonetheless, it is only applicable for volatile and semi-volatile analytes [9]. On the other hand, in HS mode, fiber coating saturation occurrence is more pronounced due to its mass transfer mechanism. On the contrary, DI mode minimizes this phenomenon and provides an improved extraction for polar species, proving its priority for biochemical and in vivo studies [10]. This is important for polar analytes, which possess low volatility and are extremely soluble in aqueous matrices [11].

Among the different strategies that have been proposed to improve the efficiency of DI-SPME, electrochemically-enhanced or electroenhanced SPME (EE-SPME) [12] is the most efficacious approach. In this method, the utilization of the electrical potential of the SPME fiber increases the migration of polar analytes toward the fiber surface. For this reason, EE-SPME not only remarkably enhances the extraction efficiency, but also improves the selectivity of separation [13]. In this sampling mode, the fiber coating is directly contacted with the sample matrix. Therefore, in addition to the general variables, sample solution conditions including sample pH and ionic strength should be controlled more carefully [14].

The most used commercial SPME fibers are polydimethylsiloxane (PDMS), polyacrylate (PA), carbowax (CW), carboxen/polydimethylsiloxane (CAR/PDMS), polydimethylsiloxane/divinylbenzene, (PDMS/DVB), carboxen/divinylbenzene (CAR/DVB) and divinylbenzene/carboxen/polydimethylsiloxane (DVB/CAR/PDMS) [15]. Nevertheless, the commercial fibers have some drawbacks such as fragility, low capacity and high cost [16]. To overcome these limitations, use of handmade SPME fibers has become widespread in recent years. In this way, different nanomaterial sorbents have been successfully used, alone or as composites [17]. These sorbents mostly have higher efficiency, compatibility, durability and lower cost in comparison with the commercial fibers [5]. In addition, very few commercial coatings are appropriate for sampling of highly polar and ionic analytes. One of the best choices to address this issue is the use of conductive polymers, especially their nanostructures [18]. Polypyrrole (PPy) is among the most popular conductive polymers for this purpose, which commonly is coated on the substrates using the electrophoretic deposition (EPD) method [19]. To prepare an SPME fiber, a thin layer of PPy can be coated on the surface of a conductive material such as a stainless steel narrow wire. In this case, the structured PPy film has a good compatibility with miniaturized analytical systems. PPy can be easily prepared from aqueous or organic solution containing a proper counter ion such as oxalate, dodecyl sulfate or perchlorate [20].

Nicotine is a toxic compound and the most important compound of tobacco, which easily absorbs into the bloodstream and is rapidly delivered to the brain. Therefore, it can cause various human diseases such as peripheral vascular disease, stroke, reproductive disorder, peptic ulcer, esophageal reflux, renal disease, enhancement of malignant hypertension, lung diseases and cancer [21]. Different analytical separation procedures such as solid-phase extraction (SPE) [22], liquid-phase microextraction (LPME) [23] and SPME [24] have been reported for sampling of nicotine in biological fluids. Nicotine is a semi-volatile polar compound, so it needs to be extracted using suitable polar fiber coatings through SPME.

Our previous experiences showed a high extraction efficiency, long durability and high chemical/mechanical resistance for the platinized stainless steel fibers coated with nanostructured PPy, for headspace sampling of nicotine from aqueous media [19]. The mentioned research was based on conventional HS-SPME. However, HS mode is generally not efficient for polar compounds. The present study is based on the DI-SPME sampling strategy, which is proper for the extraction and preconcentration of polar species such as nicotine. On the other hand, exposing the fiber to a DC voltage (in DI mode) will improve the selectivity and sensitivity of the analysis. In this research, a PPy coating of the fiber was carried out for DI-SPME and EE-DI-SPME sampling of nicotine in biological fluids, followed by gas chromatography-flame ionization detector (GC-FID) measurement. For this propose, a nanostructured PPy thin film was synthesized and simultaneously coated on the porous and cohesive surface of a platinized stainless steel wire using an amended EPD method. To the best of our knowledge, DI-SPME and EE-DI-SPME have never been reported for the sampling of nicotine. 

## 2. Results and Discussion

### 2.1. Characterization of PPy Fiber

**Figure 1:**
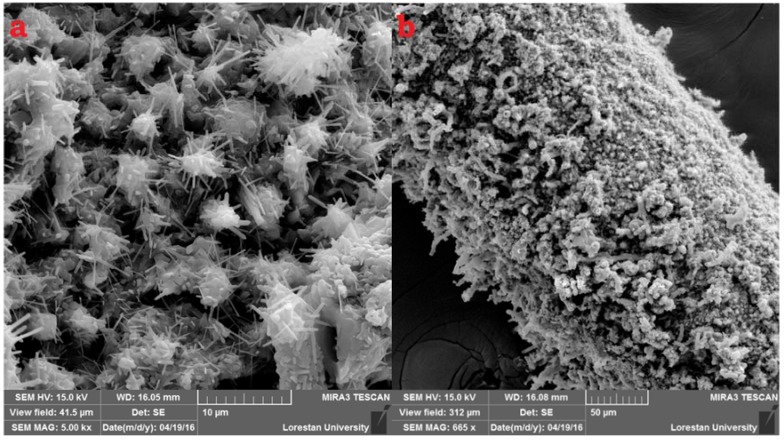
Scanning electron microscope (SEM) images of the nanostructure polypyrrole (PPy) coating at two different magnifications: (**a**) 10 µm and (**b**) 50 µm.

The morphological surface structure of the PPy-coated fiber was studied using a field-emission scanning electron microscope (FE-SEM). The SEM images of the PPy-coated platinized fiber are shown in Figure 1. It is clear that the surface of the coating is saliently porous. As depicted in Figure 1a, the nanofibers of PPy are clearly obvious. Additionally, as revealed in Figure 1b, a dense nanofibrous coating is deposited on the whole surface of the steel surface. The tubular opening of the top section can be obviously observed. Figure 1a also shows aligned PPy nanofibers vertically erecting on the steel surface.

### 2.2. Effect of Sample pH

**Figure 2:**
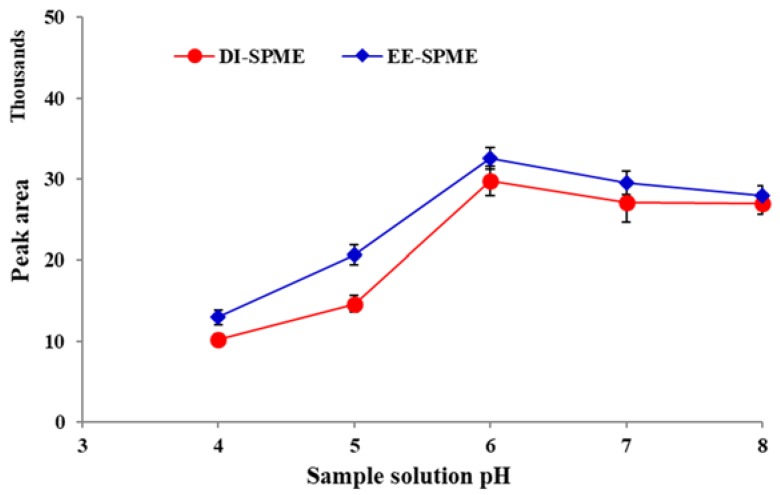
Effect of sample solution pH on the efficiency of direct immersion (DI) and electroenhanced solid phase microextraction followed by gas chromatography flame ionization detector (EE)-DI-SPME-GC-FID methods (condition: extraction temperature: 50 °C; extraction time: 20 min; stirring rate: 500 rpm; applied voltage in EE-DI-SPME: −5 V).

Nicotine exists in unprotonated, mono-protonated or di-protonated forms, depend on the acidity of the aqueous sample solution. Due to the basic properties, corresponding to the two N atoms in the structure of the unprotonated form, nicotine can obtain one or two protons in acidic solutions [25]. Unprotonated nicotine is volatile and able to enter the sample headspace, while protonated forms are not. The second nicotine acidic constant (pK_a2_) is eight, which is also the approximate dividing line between dominance by the unprotonated/protonated forms. Therefore, to obtain correct quantitative responses in the DI-DPME sampling of nicotine, the pH of the sample solution should be controlled precisely [19,26]. For this purpose, the extraction efficiency was investigated over the pH range of 4.0–8.0. As shown in Figure 2, the best extraction efficiency was achieved at pH = 6 for both the DI- and EE-DI-SPME sampling methods. The extraction efficiency decreased by using lower pHs, due to a competition between nicotine and the proton to adsorb the fiber coating.

### 2.3. Applied Voltage

**Figure 3:**
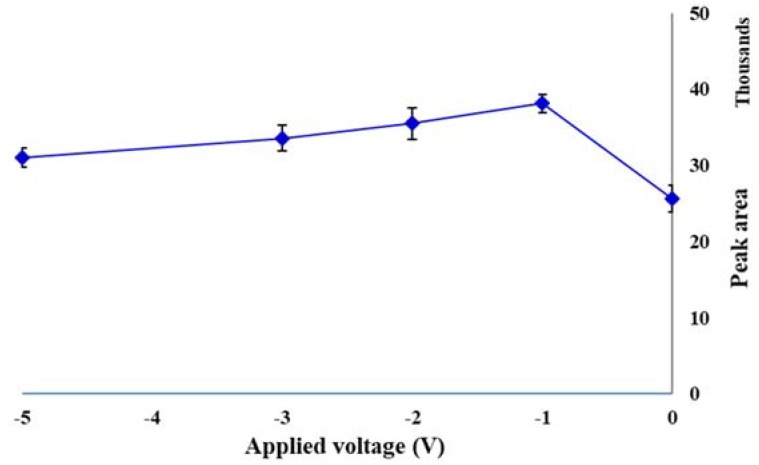
Effect of applied voltage on the extraction efficiency of the EE-DI-SPME sampling strategy (condition: sample solution pH: 6; extraction temperature: 50 °C; extraction time: 20 min; stirring rate 500 rpm).

The electric field is a new emerging driving force in various analytical sample preparation methods. It may be coupled with an established separation technique or applied as the sole driving force in a sample preparation strategy. It accelerates the mass transfer of charged species, from the sample matrix into the receiving phase (sorbent). The sample and sorbent can be either in direct contact or separated by a solid or liquid barrier. Charged species in electrolyte solutions can be forced to migrate by the applied voltage, based on their electrophoretic mobility. Imposing this kind of control on the mass transfer of charged analytes has introduced a new strategy for conducting the selective sample preparation [27]. To evaluate the effect of voltage on the performance of the EE-SPME method, first −5, −10, −15 and −20 V were evaluated. The results showed that the extraction efficiency decreased after −5 V. Therefore, the applied voltages were investigated over the range of 0–−5 V. As shown in Figure 3, the highest extraction efficiency was at −1 V and after that slightly decreased.

### 2.4. Study of Extraction Temperature and Time

**Figure 4:**
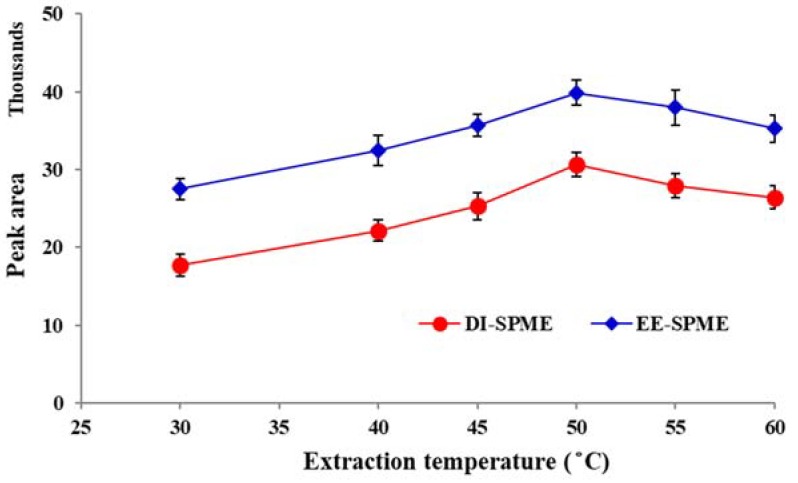
Study of the extraction temperature in the DI- and EE-DI-SPME sampling methods for the extraction of nicotine in aqueous media (condition: sample solution pH: 6; extraction time: 20 min; stirring rate: 500 rpm; applied voltage in EE-DI-SPME: −1 V).

The effect of temperature on the DI-SPME sampling is less than HS-SPME due to its mass transfer mechanism. However, to obtain an accurate quantification, it is necessary to evaluate this effect. For this propose, the effect of sample temperature was examined in the range of 20–60 °C. The results showed that the extraction efficiency increased with temperature up to 50 °C and then started to decline slightly (Figure 4). The initial rise in response can be attributed to the enhancement of migration through the diffusion layer near the fiber surface [28]. However, further raising the temperature did not favor the sorption and resulted in a decrease in the efficiency for both the DI- and EE-DI-SPME methods, due to exothermic nature of the sorption process. Hence, 50 °C was selected as the optimum value for further studies.

**Figure 5:**
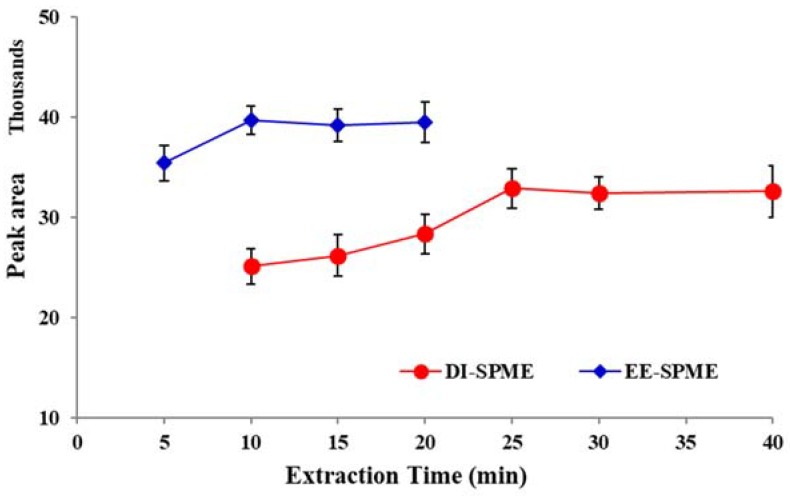
The efficiency of DI- and EE-DI-SPME-GC-FID procedures in different extraction times (condition: sample solution pH: 6; extraction temperature: 50 °C; stirring rate: 500 rpm; applied voltage in EE-DI-SPME: −1 V)

To investigate the effect of extraction time, different experiments were conducted with varying times over the range of 5–40 min. The results revealed that peak areas were increasing up to 10 min for EE-DI-SPME and up to 25 min for DI-SPME (Figure 5). Using extraction times longer than 10 and 25 min for EE-DI-SPME and DI-SPME, respectively, resulted in relatively constant peak areas. Therefore, 10 and 25 min were selected as the best extraction times for the EE-DI- and DI-SPME-GC-FID methods, respectively.

### 2.5. Effect of Stirring Rate and Ionic Strength of the Sample Solution

**Figure 6:**
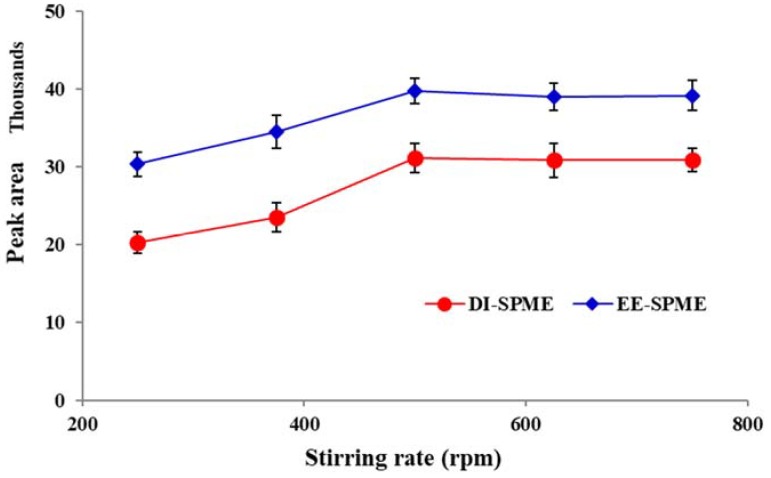
Stirring rate versus extraction efficiency for the DI- and EE-DI-SPME sampling of nicotine (condition: sample solution pH: 6; extraction temperature: 50 °C; extraction time: 20 min; applied voltage in EE-DI-SPME: −1 V).

Exposing the sample matrix to mechanical shaking, stirring, agitation, ultrasonic waves or microwave irradiation is essential to inducing stress in the sample tissue and improving the mass transfer to reduce the equilibration time. Therefore, different experiments were conducted using varying stirring rates (250–750 rpm). For both the DI- and EE-DI-SPME procedures, the extraction efficiency was increased up to 500 rpm and then remained constant (Figure 6). Thus, 500 rpm was selected as the optimum stirring rate.

To evaluate the effect of ionic strength, different amounts of NaNO_3_ (0–10% *w*/*v*) were added to the sample solution, and the EE-DI-SPME-GC-FID procedure was conducted. The results showed that increasing the ionic strength caused a gradual decrease in the extraction efficiency. A plausible explanation for this effect is the interference effect of the electrolyte ions with the electrophoretic migration of the target analyte to the fiber surface. Accordingly, for further studies, no salt was added to the sample solution.

### 2.6. Desorption Temperature and Time

**Figure 7:**
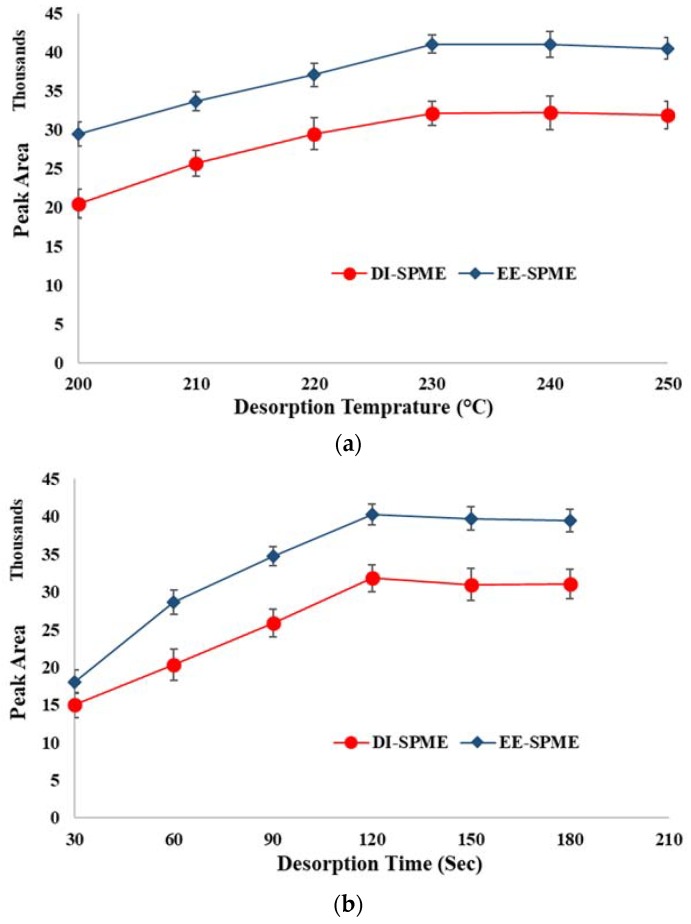
Optimization of the desorption conditions: (**a**) desorption temperature and (**b**) desorption time.

To achieve the highest analytical performance and minimize the memory effect, desorption variables were also optimized. For this purpose, the desorption temperature and time of the EE-DI-SPME-GC-FID method were varied over the ranges of 200–250 °C and 0.5–3 min, respectively. The minimum desorption time (230 °C) and temperature (2 min) that led to the highest peak areas were selected as the optimum amounts, for further experiments (Figure 7).

### 2.7. Comparison of the PPy-Coated Fiber with Commercial Fibers

The physicochemical features of the fiber coating comprise the most important variable that affects the efficiency of the SPME sampling methods. The fiber coating should be well matched with the analyte, based on its chemical properties [29]. Accordingly, PA was suggested to be the best choice for nicotine sampling, among the commercial fibers. Therefore, PA commercial fiber was compared with the developed fiber for the analysis of nicotine in aqueous media, through both the DI- and EE-DI-SPME-GC-FID methods. The obtained data demonstrated that the nanostructured PPy-coated fiber was more effective than the PA commercial fiber for both methods. It was approximately four-times more effective than PA in the EE-DI-SPME-GC-FID strategy. The prepared PPy-coated fiber was used over 120 times without any remarkable change in its extraction efficiency (<5%).

### 2.8. Method Validation

In order to investigate the analytical performances of the developed DI- and EE-DI-SPME-GC-FID procedures, their linear dynamic ranges (LDRs), limits of detection (LODs) and relative standard deviations (RSDs) were obtained under the optimum conditions. LDR, LOD and RSD of the DI-SPME-GC-FID method were found to be 0.1–10 µg mL^−1^ (*n* = 7, R^2^ = 0.995), 10 ng mL^−1^ and 6.1%, while for EE-DI-SPME-GC-FID, 0.001–8 µg mL^−1^ (*n* = 7, R^2^ = 0.998), 0.3 ng mL^−1^ and 4.6%, respectively. The relative recovery values for the analysis of 1 µg mL^−1^ nicotine were obtained as 75–105% for DI- and 91–110% for EE-DI-SPME-GC-FID. The absolute recovery for the extraction of a solution containing 1 µg mL^−1^ nicotine was found as 9.73% for DI-SPME and 12.71% for EE-DI-SPME. The volume of sorbent (V_fibre coating =_ V_fibre_ – V_fiber core_) was estimated as 1.256 µL. By considering the sample volume (5 mL), the sorbent volume (1.256 µL), absolute recovery percent (R %) and length of the coating (≈2 cm), the enrichment factors (EFs) were calculated as 38,734 and 50,597 for DI-SPME and EE-DI-SPME, respectively.

For greater elucidation of the reliability and applicability of the DI- and EE-DI-SPME-GC-FID methods, their analytical figures of merit were compared with some similar published reports [30,31,32,33,34,35]. The method applied, type of sample, LODs, LDRs and RSDs of the mentioned analytical strategies are compared in Table 1. LOD and LOQ of the developed EE-DI-SPME-GC-FID method are lower than all reported methods, except that using LC-MS (liquid chromatography mass spectrometry). Its LDR and RSD are also more acceptable than most other strategies. In addition, the analytical performances of DI-SPME-GC-FID are almost better than the presented procedures. Consequently, the priority of the proposed EE-DI-SPME-GC-FID can be given. In terms of LOD and LDR, the proposed method is not comparable with the LC-MS instrument. LC-MS is a very expensive and sensitive instrument and usually cannot be found in many research laboratories [35].

### 2.9. Analysis of Real Samples

To elucidate the functionality and applicability of the DI- and EE-DI-SPME-GC-FID procedures for real samples, they were employed for the direct analysis of nicotine in urine and plasma samples. The results (summarized in Table 2) demonstrated the reliability and applicability of the developed strategies, especially EE-DI-SPME-GC-FID, as a novel solvent-free and green analytical method, for the extraction and quantitation of nicotine in biological samples.

## 3. Materials and Methods

### 3.1. Chemicals and Materials

Nicotine (>99%) was provided by Merck chemical Co. (Darmstadt, Germany). Pyrrole (>99%) was purchased from Fluka (Buchs, Switzerland). It was redistilled and stored in a dark bottle, under nitrogen atmosphere in a fridge. Analytical reagent-grade methanol, ethanol, sodium hydroxide, sodium nitrate, lithium perchlorate and hydrochloride acid were purchased from Merck. A standard stock solution of nicotine (1000 µg mL^−1^) was prepared in methanol. The standard working solutions were prepared weekly by appropriate dilution with double-distilled water. All standard stock and working solutions were kept at 4 °C. Shirkhani Medical Laboratory (Khoramabad, Iran) kindly provided urine and plasma samples on 7 December 2016. Each of the samples was also fortified with 2 µg mL^−1^ nicotine and subjected to both procedures, three times. Urine #1 was from a 2-year-old male and Urine #2 from a 23-year-old non-smoker female. Plasma #1 was from a 47-year-old smoker male and Plasma #2 from a 38-year-old smoker male. The samples were provided with the consent of the patients and without disclosing their personal information.

### 3.2. Apparatus

For the separation and quantitation of the analyte, a Shimadzu GC-2010 Plus AF gas chromatograph (Shimadzu, Kyoto, Japan), equipped with a flame ionization detector (FID) and a split/splitless injection (SPL-2010 Plus) system, was used. Shimadzu GC solution software (Version 2.4, Shimadzu, Milan, Italy) was employed to run the instrument. The separations were accomplished by an HT-8 fused silica capillary column (25 m × 0.32 mm I.D. × 0.5 μm). Ultra-high purity-grade nitrogen (99.999%) was used as the carrier gas at a flow rate of 1 mL min^−1^. The GC was operated in the splitless mode for all SPME experiments. The injector and FID systems were maintained at 230 °C and 280 °C, respectively. The column temperature was initially set at 100 °C (1 min), then increased to 260 °C at a rate of 20 °C min**^−^**^1^ and held constant for 1 min at 260 °C. In this way, the total run time was 10 min. Hydrogen, zero-air (FID gases) and nitrogen (make-up gas) flow rates were set at 30, 300 and 30 mL min**^−^**^1^, respectively. A VEGA\\TESCAN CM120 (TESCAN, Brno, Czech Republic) field-emission scanning electron microscope (FE-SEM) was used to study the morphology of the fiber coating. Heating and stirring of the sample matrices were performed using a Heidolph MR 3001-K magnetic heater-stirrer (Kelheim, Germany). A 40-mL SPME sample vial was used as the extraction vial and a cylindrical PTFE coated magnetic stirring bar (5 mm × 2 mm O.D.) for stirring the sample solution. A 1-μL microsyringe (Hamilton, NV, USA) was used for direct injection into the GC-FID instrument. A low-cost and simple hand-made SPME fiber holder was fabricated as previously described [36] and used for handling the PPy-coated fibers.

### 3.3. Preparation of the Fiber Substrate and Its Coating Using the EPD Method

Due to direct contact with the sample solution in DI-SPME, the fibers usually become soft and soggy. On the other hand, the fiber coatings often become fragile and unstable, after a few times of use. The main reason is the smoothness of the coated substrates, which prevents firm attachment of the coating material to the fiber’s bed. This issue has been mainly addressed by platinizing the surface of stainless steel fibers [5]. This created a porous, cohesive and large surface area bed and consequently resulted in a highly porous, durable and chemically-/mechanically-resistant fiber. Therefore, the surface of a stainless steel narrow wire (0.1 mm O.D., 4 cm L) was platinized using an amended EPD method. The prepared wire was electrochemically coated by PPy using a two-electrode system and lithium perchlorate as the counter ion, as described previously [19].

### 3.4. Extraction Procedure

For DI-SPME sampling, 5 mL of the sample solution (containing 1 µg mL^−1^ nicotine at pH = 6) were transferred into a 40 mL SPME vial. The SPME vial was placed into a water bath at 50 °C, along with stirring at 500 rpm. Then, the SPME fiber was directly inserted into the sample solution. After 25 min for complete equilibration, the fiber was retracted into the fiber holder and immediately injected into the GC-FID instrument for thermodesorption of the analyte at 230 °C for 2 min. For the EE-DI-SPME sampling, all conditions were similar except for applying DC voltage and extraction time. For this purpose, the needle of the SPME fiber holder and a platinum wire were connected to the DC power supply, and a −1 V DC potential was applied. After 10 min, the fiber was pulled out and injected into GC-FID.

## 4. Conclusions

The surface of a narrow stainless steel wire was made porous and cohesive and was coated with nanostructured polypyrrole using an amended EPD procedure. Then, the two DI- and EE-DI-SPME-GC-FID strategies were developed and compared by using the PPy-coated fiber. Based on the obtained results, the EE-DI-SPME method showed lower LOD and RSD and wider LDR for the extraction and determination of nicotine in aqueous media. In addition, the equilibrium time between the fiber coating and sample solution was shorter for EE-DI-SPME, due to reinforced-mass transfer caused by the DC voltage driving force. The prepared fiber indicated substantial durability, a longer lifetime and higher extraction efficiency for nicotine compared with PA commercial fiber in both methods.

## Figures and Tables

**Table 1:** Comparison of the developed DI- and EE-DI-SPME-GC-FID methods with similar reported procedures for the quantification of nicotine in liquid samples. LDR, linear dynamic range.

Method	Sample	LDR (μg mL^−1^)	LOD (ng mL^−1^)	RSD (%)	Ref.
LE-PTC-GC-MS	Urine, saliva and hair	0.01–3	0.60	3–20	[30]
MISPE-UV	Spiked urine	0.17–9.7	NR	10	[31]
UE-GC-FID	Pharmaceutical formulations	1–500	250	2	[32]
LC-UVDAD	Human milk	NR	13	5.8	[33]
GC-FID	Aqueous extract of tabacum leaves	5–60	500	3.2	[34]
IT-SPME-LC-MS	Human urine and saliva	0.0005–0.02	0.015–0.04	4.7–11.3	[35]
DI-SPME-GC-FID	Urine and plasma	0.1–10	10	6.1	This work
EE-DI-SPME-GC-FID	Urine and plasma	0.001–10	0.3	4.6	This work

LE-PTC-GC-MS: liquid extraction into dichloromethane through a phase-transfer catalyst (for derivatization) followed by GC-MS determination. MISPE-UV: molecularly-imprinted solid-phase extraction method coupled to spectrophotometric determination. UE-GC-FID: ultrasonic extraction with heptane followed by GC-FID separation and quantitation. LC-UVDAD: liquid chromatography-diode array UV detection. IT-SPME-LC-MS: in-tube SPME coupled with liquid chromatography-mass spectrometry. NR: not reported. LOD: limit of detection.

**Table 2:** Application of the developed DI- and EE-DI-SPME-GC-FID procedures for the extraction and measurement of nicotine in biological fluids of the human body.

Sample	Added (µg mL^−1^)	DI-SPME-GC-FID		EE-SPME-GC-FID	
		µg mL^−1^	R (%)	µg mL^−1^	R (%)
Urine 1 (Male, 2 years)	0	NF	---	NF	---
	2	1.82 (8.6)	90	2.20 (5.1)	110
Urine 2 (Female, non-smoker, 23 years)	0	NF	---	NF	---
	2	1.88 (7.2)	94	2.06 (4.9)	103
Plasma 1 (Male, smoker, 47 years)	0	1.43 (9.5)	---	1.95 (7.4)	---
	2	3.57 (6.3)	105	3.61 (3.8)	91
Plasma 2 (Male, smoker, 38 years)	0	2.1 (5.8)	---	2.47 (4.5)	---
	2	3.03 (4.7)	75	4.4 (3.6)	98

R: recovery. NF: not found.
